# Protective effect of mild endoplasmic reticulum stress on radiation-induced bystander effects in hepatocyte cells

**DOI:** 10.1038/srep38832

**Published:** 2016-12-13

**Authors:** Yuexia Xie, Shuang Ye, Jianghong Zhang, Mingyuan He, Chen Dong, Wenzhi Tu, Peifeng Liu, Chunlin Shao

**Affiliations:** 1 Institute of Radiation Medicine, Fudan University, Shanghai, China; 2 Central Laboratory, Renji Hospital, School of Medicine, Shanghai Jiaotong University, Shanghai, China

## Abstract

Radiation-induced bystander effect (RIBE) has important implications for secondary cancer risk assessment during cancer radiotherapy, but the defense and self-protective mechanisms of bystander normal cells are still largely unclear. The present study found that micronuclei (MN) formation could be induced in the non-irradiated HL-7702 hepatocyte cells after being treated with the conditioned medium from irradiated hepatoma HepG2 cells under either normoxia or hypoxia, where the ratio of the yield of bystander MN induction to the yield of radiation-induced MN formation under hypoxia was much higher than that of normoxia. Nonetheless, thapsigargin induced endoplasmic reticulum (ER) stress and dramatically suppressed this bystander response manifested as the decrease of MN and apoptosis inductions. Meanwhile, the interference of BiP gene, a major ER chaperone, amplified the detrimental RIBE. More precisely, thapsigargin provoked ER sensor of PERK to initiate an instantaneous and moderate ER stress thus defensed the hazard form RIBE, while BiP depletion lead to persistently destroyed homeostasis of ER and exacerbated cell injury. These findings provide new insights that the mild ER stress through BiP-PERK-p-eIF2α signaling pathway has a profound role in protecting cellular damage from RIBE and hence may decrease the potential secondary cancer risk after cancer radiotherapy.

Since Nagasawa and Little^1^ first reported the phenomenon that sister chromatid exchanges could be generated in unirradiated cells after receiving signals from irradiated cells, considerable evidence has accumulated in support of the substantial existence of radiation induced bystander effect (RIBE). Growing documents have demonstrated that bystander responses could be regulated by two widely accepted models including gap junction intercellular communication (GJIC)^2^ and soluble molecules secreted from irradiated cells mediated inflammatory responses^3^. Numerous biological endpoints have been observed in RIBE, such as sister chromatid exchanges^1^, genomic instability^4^, DNA methylation^5^, apoptosis^6^, malignant invasiveness^7^ and terminal differentiation^8^.

To date, a large variety of signaling molecules have been proved as pivotal bystander modulators including free radicals^9^,^10^,^11^, calcium flux^12^, interleukins^13^, cytochrome-c^11^,^14^, cAMP^15^, transforming growth factors-β1 (TGF-β1)^16^, tumor necrosis factor-α (TNF-α)^17^, nuclear factor kappa B (NF-κB)^18^ and MAPK^18^,^19^. In addition, most previous studies of RIBE have focused on these signaling molecules transferring from irradiated cells toward non-irradiated bystander cells. Recent investigations reported that after sensing the bystander exposures, the non-irradiated cells could also send feedback signals to their neighboring irradiated cells^15^,^18^. Therefore, irradiated tumor tissue and cells can trigger bystander responses to adjacent tumor cells, and hence exacerbate radiation injury and amplify the efficacy of cancer therapy.

However, extensive *in vitro* and *in vivo* studies have demonstrated that in response to irradiation, cancer cells could also evoke bystander responses to normal tissue and cells^10^,^13^,^20^, which may enhance the occurrence of the secondary cancer risk after radiotherapy. On the other hand, it was reported that bystander responses could induce differentiation of primary cells and have a protective role in removing potentially damaged cells in response to low dose irradiation and then decrease radiation cancer risk^8^. Thus, elucidating the defense and self-protective mechanisms of bystander normal cells would be crucial for better understanding of overall cancer risk control.

Hepatocellular carcinoma is one of the most prevalent cancers worldwide and has become the leading cause for cancer-related deaths in China, as it commonly develops local hypoxic regions, which are closely associated with radioresistance for augmentation post-irradiation molecular restoration. Meanwhile, our recent investigation showed that as critical mediator of radiation-induced DNA damage, ROS was more effective in hypoxic hepatoma cells than normoxic cells^10^. Furthermore, our previous study revealed that the fraction of bystander micronuclei (MN) formation in the yield of radiation-induced MN under hypoxic condition was much higher than that under normoxic condition^21^. Thus, the bystander hepatocyte cells would receive more damage signals from irradiated hepatoma cells during radiotherapy.

The endoplasmic reticulum (ER) is a multifunctional organelle that participates in a variety of signaling pathways for the maintenance of organismal and cellular function and survival^22^. This process is tightly supervised by an ER-resident chaperone, termed as immunoglobulin heavy chain binding protein (BiP), which is in charge of maintaining proteins in folding-competent state, ER calcium homeostasis, as well as monitoring the accumulation of unfolded/misfolded proteins^23^. Physiological and/or environmental perturbations of ER homeostasis is known as ER stress to trigger the activation of unfolded protein response (UPR)^24^. Accumulated evidence suggests that instantaneous or moderate ER stress protects cells from injury, while persistent or severe ER stress induces cell apoptosis and death and hence removes seriously damaged cells to decrease cancer risk^25^. Although the function of ER has been deeply investigated in direct physiological and environmental stress, the underlying molecular mechanism of ER stress in response to RIBE remains unknown and has not been reported in literature. The present study investigated the role of ER stress of hepatocyte cells in the bystander responses induced by irradiated hepatoma cells under normoxic and hypoxic conditions, and found that the moderate ER stress was beneficial for bystander hepatocyte cells to defense against detrimental RIBE from hepatoma cells in alleviating cell injury including DNA damage and cellular apoptosis.

## Results

### RIBE between hepatoma and hepatocyte cells at different oxygen status

**Figure 1:**
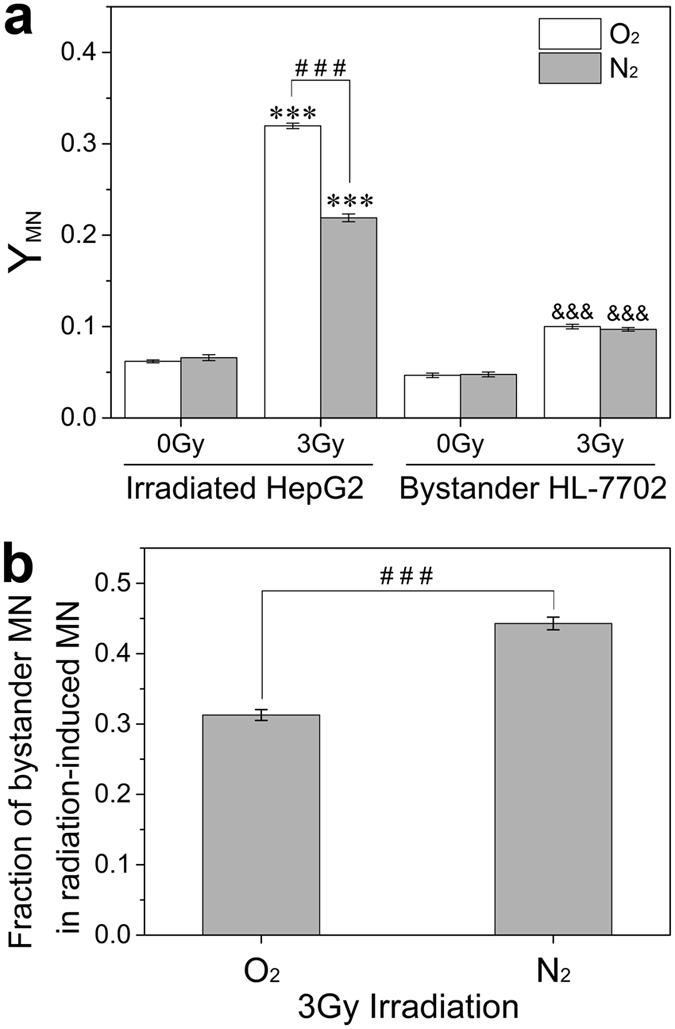
The yields of MN (Y_MN_) in irradiated HepG2 cells and bystander HL-7702 cells at different oxygen status (**a**) and the ratio of bystander MN of HL-7702 cells to the radiation-induced MN of hepatoma cells at different oxygen status (**b**). ***P < 0.001 compared to non-irradiated control of HepG2 cells; ^&&&^*P* < 0.001 compared to non-irradiated control of HL-7702 cells; ^###^*P* < 0.001 between the indicated groups.

Our previous study has shown that RIBE plays a more important role in radiation damage of hepatoma cells under hypoxia than normoxia^21^, but it is unclear whether this special contribution of bystander effect exists between hepatoma cells and hepatocyte. Figure 1a showed that, after 3 Gy irradiation, the yield of MN in hepatoma HepG2 cells was obviously increased but it was dramatically degraded under hypoxia. Meanwhile, radiation-induced bystander MN formations in hepatocyte HL-7702 cells were obviously enhanced under both hypoxia and normoxia, but the level of this bystander DNA damage was independent of oxygen status. Thus, the unit quantitative irradiated cells under hypoxia release more signaling factors conferring serious DNA damage than that under normoxia. Accordingly, it can be calculated that the ratio of the bystander MN induction in HL-7702 cells to the yield of radiation-induced MN formation in HepG2 cells under hypoxia is much higher than that under normoxia (Fig. 1b), indicating that the irradiated hepatoma cells under hypoxia play more profound role in radiation-induced bystander damage to neighboring hepatocyte cells.

### ER stress was involved in RIBE

**Figure 2:**
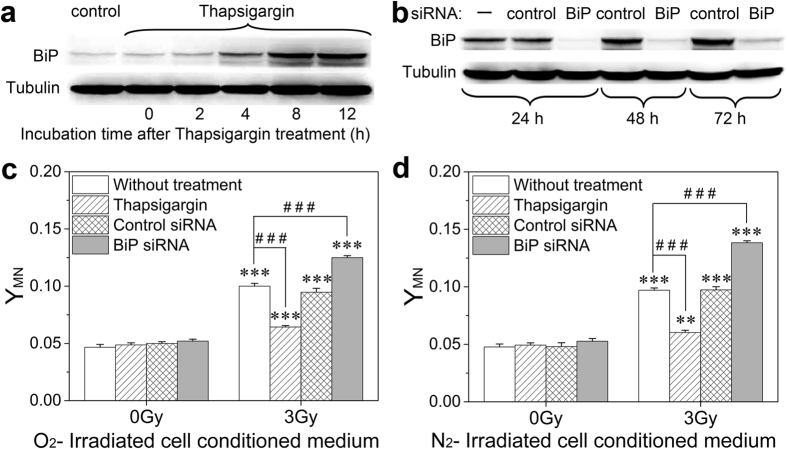
Influence of endoplasmic reticulum stress response on bystander HL-7702 cells. (**a**) Time course of BiP expression in HL-7702 cells after thapsigargin treatment. (**b**) Expression of BiP protein in HL-7702 cells transfected with BiP siRNA or its control, respectively. “–”, without siRNA treatment. (**c**,**d**) The yields of MN formation in HL-7702 cells treated with ICCM from normoxic (**c**) and hypoxic (**d**) HepG2 cells. Before receiving ICCM, HL-7702 cells were pretreated with 0.5 μM thapsigargin for 1.5 h, transfected with BiP siRNA or its control, respectively. **P < 0.01, ***P < 0.001 compared to non-irradiated control; ^###^*P* < 0.001 between the indicated groups.

ER is a principal mediator in cell signal transduction, and disruption of its normal function (a mechanism known as ER stress) could associate with DNA damage and apoptosis^26^. To explore whether ER stress is involved in the RIBE between irradiated HepG2 cells and bystander HL-7702 cells, we treated HL-7702 cells with thapsigargin (an ER stress activator) and BiP siRNA for initiating or disturbing the ER stress. Figure 2a illustrates that the expression of BiP protein, an ER stress marker, in HL-7702 cells was gradually increased over time after thapsigargin treatment for 1.5 h and had a peak value at 8 h then became relatively stable up to 12 h. Conversely, the treatment of cells with BiP siRNA clearly decreased the expressions of BiP protein at 24–72 h post-transfection (Fig. 2b).

Further measurement illustrates that the above bystander response induced by the irradiated HepG2 cells was markedly suppressed when the HL-7702 cells were pretreated with the ER stress activator of thapsigargin but, in opposite, was enhanced when the HL-7702 cells were pretreated with the BiP siRNA (Fig. 2c and d). These results suggest that the ER stress may protect HL-7702 cells against harmful bystander signaling factors released from irradiated HepG2 cells.

### Expression of the ER stress marker BiP in the bystander cells

**Figure 3:**
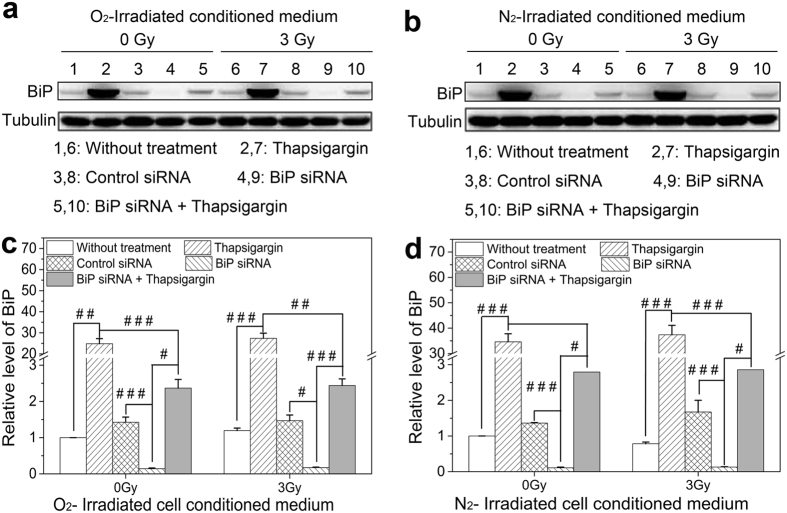
Induction of BiP in bystander HL-7702 cells treated with thapsigargin, BiP siRNA, or their combination before receiving ICCM from normoxic and hypoxic HepG2 cells. (**a,b**) Representative protein images of the cells treated with normoxic and hypoxic ICCM, respectively. (**c,d**) Relative expression level of BiP protein in the cells treated with normoxic and hypoxic ICCM, respectively. ^#^*P* < 0.05, ^##^*P* < 0.01, ^###^*P* < 0.001 between the indicated groups.

BiP is an essential ER chaperone and acts as the main marker responsible for ER stress^27^. In human cancer tissues, such as liver, melanoma, breast and colon, BiP has positive correlation with cell proliferation, survival and tumor progression^28^. To confirm the participation of BiP in RIBE, we measured the protein expression of BiP in the bystander HL-7702 cells that had been treated with the irradiated cell conditioned medium (ICCM) generated from normoxic or hypoxic HepG2 cells. Figure 3 showed that the expression of BiP was extensively increased in the thapsigargin treated cells but was significantly suppressed in the BiP siRNA-transfected cells. However, when the BiP was interference, the exogenous thapsigargin still enhanced the expression of BiP protein but with a lower level compared with that in the thapsigarin-treated HL-7702 cells, indicating that the activation effect of thapsigargin in the ER stress response was mainly blocked by the absence of BiP protein and that BiP plays a crucial role in the ER stress. Moreover, the expression of BiP in the thapsigargin treated cells was higher under hypoxic ICCM treatment than that of normoxia, suggesting that the ER stress initiated HL-7702 liver cells are more sensitive to irradiated hypoxia hepatoma cells.

### Influence of ICCM on BiP-PERK-p-eIF2α pathway in the bystander cells

**Figure 4:**
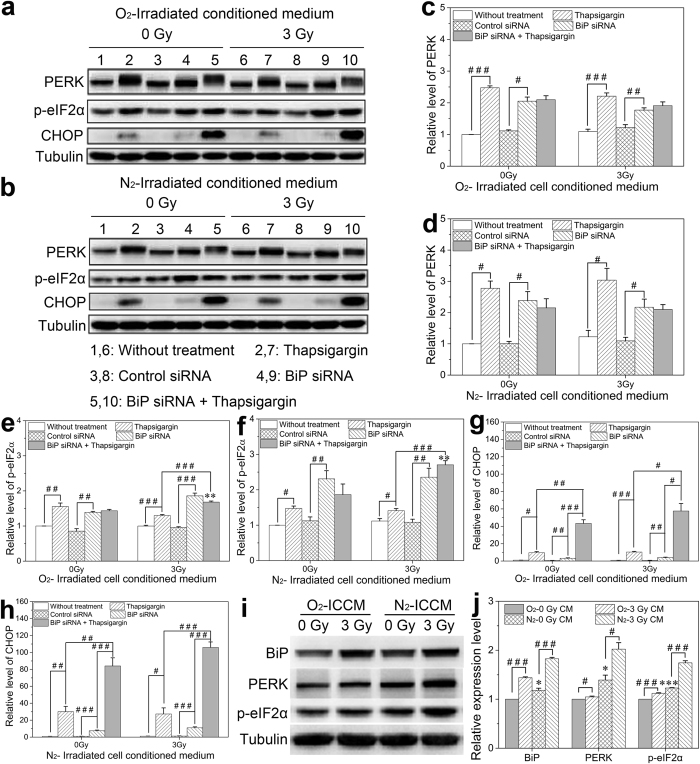
Induction of the PERK signaling factors in bystander HL-7702 cells treated with thapsigargin, *BiP* siRNA, or their combination before receiving ICCM from normoxic and hypoxic HepG2 cells. (**a,b,i**) Representative protein images in the cells treated with normoxic and hypoxic ICCM, respectively. (**c,d,e,f,g,h**) Relative expression levels of PERK, p-eIF2α and CHOP protein in the cells treated with normoxic and hypoxic ICCM, respectively. (**j**) Relative expression levels of BiP, PERK and p-eIF2α protein in the cells treated with normoxic and hypoxic ICCM, respectively. *P < 0.05, **P < 0.01 compared to non-irradiated control; ^#^*P* < 0.05, ^##^*P* < 0.01, ^###^*P* < 0.001 between the indicated groups.

PERK, pancreatic ER eukaryotic translation initiation factor 2 (eIF2a) kinase, is a type-I transmembrane protein with a cytosolic Ser/Thr kinase domain. During ER stress, the luminal domain of PERK dissociates the BiP binding and dimerizes in the plane of ER membrane, leading to trans-autophosphorylation and activation of its kinase activity. The protein expressions of this PERK-p-eIF2α pathway in the bystander cells were detected with Western blotting assay (Fig. 4a and b). As shown in Fig. 4c and d, both thapsigargin treatment and BiP interference obviously increased the expression of PERK protein. The combination treatment of thapsigargin plus BiP interference also increased the content of PERK but without significant differences compared with that of either thapsigargin or BiP interference alone. These PERK expression alterations after different treatment were not influenced by the oxygen condition and irradiation. Nevertheless, the function of PERK is determined by its kinase activity. As eIF2α is a major downstream effector of the PERK-mediated ER stress response, we then assessed the phosphorylation level of eIF2α.

The results of Western blotting assay in Fig. 4e and f revealed that both thapsigargin treatment and BiP interference promoted the phosphorylation level of eIF2α. However, compared to the thapsigargin treatment, BiP interference significantly increased the expression of p-eIF2α protein, indicating that the up-regulation of PERK protein expression did not mean its activity being increased. In addition, compared to the non-irradiated cells, the conditioned medium from irradiated cells markedly augmented the expression of p-eIF2α in the bystander cells treated with BiP interference plus thapsigargin, and it was much higher than that of thapsigargin treatment group, especially under hypoxia condition. Therefore, the irradiated hepatoma cells could induce bystander effect by triggering the ER stress response in the bystander cells, and this bystander effect in hypoxic cells is stronger than in normoxic cells.

We further detected the expression of CHOP, a downstream factor of eIF2α, and found that it was enhanced in the bystander cells under both thapsigargin treatment and BiP interference, but the effect of thapsigargin was more conspicuous than BiP interference (Fig. 4g and h). In contrast, the combination treatment of thapsigargin and BiP interference extensively elevated the level of CHOP protein, and the proportion of up-regulated CHOP in the bystander cells treated with hypoxic conditioned medium was much more notable than that of normoxic treatment group. These observations proved that the PERK–p-eIF2α–CHOP pathway was involved in the RIBE.

To concretely validate whether this is a self-defense mechanism in bystander hepatocyte cells against the damage responses from irradiated hepatoma cells, we further investigated the expression levels of BiP-PERK-p-eIF2α signaling pathway in bystander cells before and after DNA damage. Figure 4i and j clearly showed that 0 Gy-conditioned medium from hypoxia cells obviously promoted the expressions of BiP, PERK and p-eIF2α than that from normoxia cells. Importantly, compared with 0 Gy-conditioned medium, the expressions of BiP, PERK and p-eIF2α in the bystander cells treated with 3 Gy-conditioned medium was increased by 55%, 45% and 42% under hypoxia, and 44%, 5%, and 12% under normoxia, respectively. These results demonstrated that the BiP-PERK-p-eIF2α pathway played much stronger effect under hypoxia condition.

### Influence of ICCM on BiP-ATF6-XBP-1 pathway in the bystander cells

**Figure 5:**
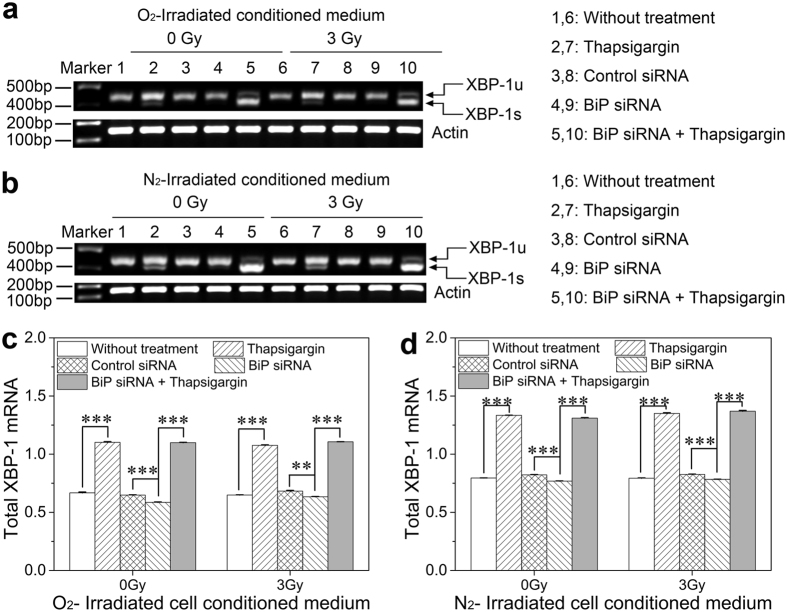
Induction of XBP-1 mRNA in bystander HL-7702 cells treated with thapsigargin, BiP siRNA, or their combination before receiving ICCM from normoxic and hypoxic HepG2 cells. (**a,b**) Typical mRNA image of XBP-1 in the cells treated with normoxic and hypoxic ICCM, respectively. (**c,d**) Expression level of XBP-1 mRNA in the cells treated with normoxic and hypoxic ICCM, respectively. **P < 0.01, ***P < 0.001 between the indicated groups.

In the stressed ER, ATF6 could be released from the chaperone BiP and translocated to the Golgi apparatus that is sequentially cleaved to activate the transcription of XBP-1. The mRNA assay showed that the thapsigargin pretreatment significantly enhanced the total expression of XBP-1 mRNA, while BiP interference decreased the mRNA level of XBP-1 (Fig. 5). However, the thapsigargin treatment also promoted the increase of XBP-1 mRNA in the BiP interference cells, indicating that cells with BiP interference failed to restore ER homeostasis, which led to the excessive reaction of ER stress and resulted in cell injury. In addition, the expression level of XBP-1 mRNA was much higher under hypoxic ICCM treatment than that of normoxia, confirming that hypoxic hepatoma cells are more likely to induce bystander effects. Nevertheless, the expression level of XBP-1 mRNA showed no appreciable changes between 0 Gy and 3 Gy ICCM treatment both under normoxic and hypoxic conditions, suggesting that the ATF6-XBP-1 mRNA pathway plays a critical role in thapsigargin and BiP siRNA treatment, but is not responsible for RIBE.

### Influence of ICCM on BiP-IRE1α splicing pathway in the bystander cells

**Figure 6:**
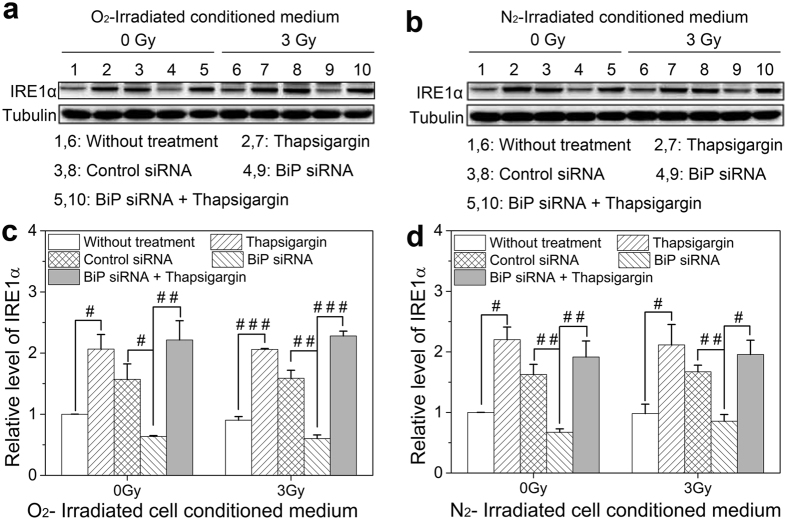
Induction of the IRE1α protein in bystander HL-7702 cells treated with thapsigargin, BiP siRNA, or their combination before receiving ICCM from normoxic and hypoxic HepG2 cells. (**a,b**) Representative protein images in the cells treated with normoxic and hypoxic ICCM, respectively. (**c,d**) Relative expression level of IRE1α protein in the cells treated with normoxic and hypoxic ICCM, respectively. ^#^*P* < 0.05, ^##^*P* < 0.01, ^###^*P* < 0.001 between the indicated groups.

On the ER stress, IRE1α is released from BiP and dimerizes in the plane of the ER membrane, leading to transautophosphorylation and activation of its kinase and RNase activities which splices and encodes XBP-1 mRNA to form active transcription factors XBP-1s (spliced XBP-1, XBP-1s). It was measured that the thapsigargin pretreatment markedly increased the expression of IRE1α, while the BiP interference suppressed the content of IRE1α (Fig. 6), indicating that the thapsigargin treatment induced ER stress in HL-7702 cells and activated IRE1α pathway, but the BiP interference inhibited this process. The thapsigargin treatment also upregulated the expression of IRE1α in the BiP interfered cells, suggesting that the thapsigargin treatment could directly promote the expression of IRE1α to protect cells from damage. However, the expression of IRE1α protein presented negligible differences between 0 Gy and 3 Gy ICCM treatment both under normoxic and hypoxic conditions. Since IRE1α is a kinase and possesses RNase activity for XBP-1 mRNA splicing, we further evaluated the XBP-1 mRNA splicing.

As displayed in Fig. 5 and Table 1, in response to thapsigargin treatment, the increased IRE1α dramatically promoted XBP-1 mRNA to splice into XBP-1s. However, the BiP interference could not induce XBP-1 mRNA splicing, indicating that the XBP-1 mRNA activity was inhibited at ER homeostasis. But when ER stress is excited, the XBP-1 mRNA was up-regulated to protect cells from external injury. Similar to total amount of XBP-1 mRNA, the BiP interference significantly augmented the formation of active XBP-1 mRNA in the thapsigargin-treated cells. Nonetheless, XBP-1 mRNA splicing also had no differences between 0 Gy and 3 Gy ICCM treatment both under normoxic and hypoxic conditions. These results verified that, in the BiP interference cells, thapsigargin could trigger abnormal response of ER stress leading to cell injury, but this process did not account for RIBE.

### ER stress protects bystander cells against apoptosis

**Figure 7:**
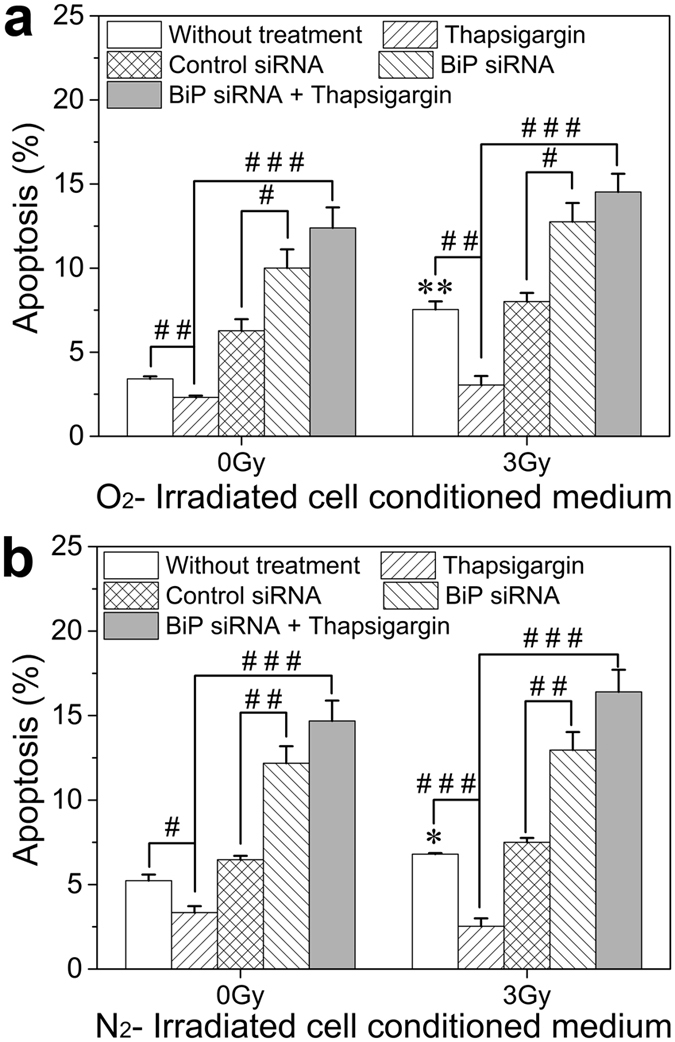
Induction of apoptosis in bystander HL-7702 cells treated with thapsigargin, BiP siRNA, or their combination before receiving ICCM from normoxic and hypoxic HepG2 cells. (**a**) Fraction of apoptosis in the cells treated with normoxic ICCM. (**b**) Fraction of apoptosis in the cells treated with hypoxic ICCM. *P < 0.05, **P < 0.01 compared to non-irradiated control; ^#^*P* < 0.05, ^##^*P* < 0.01, ^###^*P* < 0.001 between the indicated groups.

Clearly, the above results revealed that the BiP-PERK-p-eIF2α signaling pathway participated in RIBE, which would contribute to cellular apoptosis. Concordantly, the severe or persistent ER stress could lead to apoptosis. We therefore studied the role of instantaneous ER stress in apoptosis induction of bystander cells. Figure 7 showed that the ICCM from both normoxic and hypoxic hepatoma cells could induce apoptosis in the bystander cells, which was obviously depressed by the thapsigargin pretreatment but was extremely enhanced by the BiP interference. These data, consistent with the results of MN formation, further provided evidence indicating that the irradiated hepatoma cells could promote bystander apoptosis in hepatocyte cells and this effect could be ablated by instantaneous ER stress.

## Discussion

Most previous studies of RIBE have paid close attention on the bystander signals transmitted from irradiated cells to non-irradiated bystander cells. Recently, the defense system of bystander cells responding to harmful signals has received much attention^29^,^30^,^31^. Our present work disclosed that the bystander hepatocyte cells could trigger innate ER stress against the damage responses from irradiated hepatoma cells.

ER is a highly dynamic and multifunctional organelle responsible for synthesizing and packaging proteins as well as signaling processes. Perturbations of ER function lead to the accumulation of unfolded and misfolded proteins in the ER lumen (a condition called ER stress) and activate the UPR. The moderate ER stress aims at restoring cellular homeostasis and alleviating damage, however, the persistent and severe ER stress induce cell apoptosis and death^25^. Our results provided novel evidence that the instantaneous ER stress contributed to the resistance of hepatocyte cells to bystander DNA damage and apoptosis induced by the ICCM from hepatoma cells. While depletion of the principal ER stress mediator of BiP amplified the harmful bystander effects, which was much conspicuous under hypoxia than that of normoxia. These results were consistent with previous findings that the ER stress-primed mesangial cells with thapsigargin treatment not only became insensitive to IL-1β and TNF-α, but also substantially suppressed their own immune activation induced by LPS-activated macrophages^32^. At this point, the self-defense machinery may play an important role in the bystander response.

PERK is one of the crucial ER stress transducers. In the ER-unstressed status, PERK is in a complex containing BiP that attenuates PERK activity. However, when the cells are subjected to a stress, leading to the imbalance of ER homeostasis, PERK can be dissociated from BiP, resulting in its autophosphorylation and activation^33^. Thus, the enhanced expression of PERK protein and its phosphorylation can be used as a marker of ER stress. In this study, we showed that the thapsigargin pretreatment significantly enhanced the expression of PERK protein and stimulated ER stress.

In addition, eIF2α is a key substrate of PERK and it can be directly phosphorylated by active PERK. Similarly, we found that the thapsigargin pretreatment extensively promoted the expression of p-eIF2α correlating with the decrease of MN formation and apoptosis induction in the bystander cells, which sufficiently supports the concept that thapsigargin stimulates ER stress and activates p-eIF2α through PERK pathway and hence to relieve radiation-induced bystander chromosome damage and aid cell survival via decreasing the load of nascent proteins in the ER^25^. Consistently, Rahmani *et al*.^34^ reported that inhibition of the activity and expression of PERK protein significantly depressed the phosphorylation of eIF2α and amplified the detrimental effects of Sorafenib-induced cell apoptosis. Several studies also revealed that eIF2α could be phosphorylated by thapsigargin and tunicamycin^35^,^36^, which in turn enhanced the lethal effect when suspension-activated PERK^35^. Recent reports demonstrated that the persistent PERK signaling protected cells from anoikis^37^, and the intact PERK-eIF2α pathway could defense against cigarette smoke extract insult in HBE cells^38^. These studies further supported our findings that the PERK-eIF2α pathway is crucial for hepatocyte cell survival under the exposure of bystander signals.

In contrast, the thapsigargin treatment in the BiP depletion cells robustly promoted the expression of p-eIF2α, which seems to be contradictory with the data of thapsigargin treatment alone. Nevertheless, several studies have reported that when the stress was excessive or unable to resolve, p-eIF2α could inhibit cell cycle progression^39^,^40^,^41^. Additionally, the sustained phosphorylation of eIF2α could not protect beta-cells against free fatty acids-mediated apoptosis but exacerbated free fatty acid-induced dysfunction and apoptosis^42^. These is consistent with our findings that the BiP interference persistently destroyed the homeostasis of ER and lead to cell apoptosis. Thus, the thapsigargin treatment aggravated BiP-depletion-induced cell responses and resulted in apoptosis promotion associated with the over expression of CHOP protein^43^.

With respect to the roles of other ER stress effectors of IRE1α and ATF6 in RIBE, our results showed that IRE1α was obviously increased after thapsigargin treatment but attenuated since BiP depletion after receiving the ICCM from irradiated hepatoma cells. Accordingly, as the downstream of ATF6, XBP-1 mRNA had similar tendency with IRE1α in the stress response. It has been previously shown that during the ER stress, the activation of IRE1α cleaves XBP-1 mRNA into XBP-1s^44^, which is involved in a wide range of signaling cascades including ER chaperones, ER biogenesis, protein trafficking and inflammatory responses^28^. As expected, in this study, the proportion of XBP-1s was considerably enhanced along with the up-regulation of IRE1α but was not detected in the BiP depletion cells. However, the expression of IRE1α protein and XBP-1 mRNA splicing had no significant differences between 0 Gy and 3 Gy ICCM treatment groups both under normoxic and hypoxic conditions. These results suggest that the ER stress can be triggered by promoting IRE1α to splice ATF6-induced XBP-1 mRNA into XBP-1s, which could be a potential protective factor of cell apoptosis. While on bystander exposures, the BiP-ATF6-XBP-1 and BiP-IRE1α splicing signaling arms did not participate in RIBE.

Taken together, this study disclosed that the ER stressor could protect hepatocyte cells from damage induced by irradiated bystander hepatoma cells. The present work also defines a novel mechanism that a moderate ER stress may be beneficial for hepatocyte cells to defense against detrimental RIBE from hepatoma cells as it can alleviate cell injury including DNA damage and cellular apoptosis. Considering our previous findings of an increased radiosensitivity in SirT1-deficient hepatoma cells under both normoxia and hypoxia^45^, a reasonable clinical treatment strategy can be suggested to improve the efficiency of radiotherapy by intervening SirT1 gene expression in hepatocellular carcinoma to enhance its radiosensitivity as well as moderating the activation of ER stress in hepatocytes to protect normal cells.

## Methods

### Cell culture and hypoxic incubation

HepG2 hepatoma cells and HL-7702 hepatocyte cells were obtained from the Shanghai Cell Bank of China. HepG2 cells were maintained in DMEM medium (HyClone, Beijing, China) containing 4.5 g/L glucose and 4 mM glutamate. HL-7702 cells were grown in RPMI 1640 medium (HyClone). Both culture media contained 100 U/mL penicillin, 100 μg/mL streptomycin and 10% FBS (Gibco Invitrogen, Grand Island, NY, USA). All cultures were maintained at 37 °C in a fully humidified incubator with 5% CO_2_, where the oxygen tension was held at either 21% (normoxia) or 1% (hypoxia).

### Cell irradiation and medium transfer experiments

HepG2 cells were seeded onto 35-mm Petri dish (1 × 10^5^) and grew for 1 day. For the hypoxia experiment, after 12 h of hypoxic treatment, the cell dishes sealed in a box filled with N_2_ gas were irradiated at room temperature with γ-rays at a dose rate of 0.79 Gy/min using a ^137^Cs irradiator (Gammacell-40, MDS Nordion, Toronto, Ontario, Canada). Immediately after irradiation, the HepG2 cells were washed triply with hypoxic PBS in an airtight hypoxic bench (Model YQX-1, Shanghai Yuejin Mdeical Instruments, Shanghai, China) then maintained with fresh hypoxic medium and incubated in the hypoxic incubator for further 2 h to prepare the conditioned medium. For the normoxia experiment, HepG2 cells were treated in the same way but under normoxia. Nonirradiated control samples were treated in the same way except for irradiation. After incubation, the irradiated cell conditioned medium (ICCM) was harvested and filtered through a 0.22 μm filter and transferred to a 35-mm Petri dish where 1 × 10^5^ HL-7702 bystander cells had been incubated for 1 day at that time. HL-7702 cells were treated with the ICCM for 4 h and then harvested for further analysis.

### Chemical treatment and transfection

In some experiments, HL-7702 cells were treated with 0.5 μM thapsigargin (Sigma, St. Louis, MO, USA) for 1.5 h, then immediately washed triply with PBS before ICCM treatment. Thapsigargin is a non-competitive inhibitor of Ca^2+^-ATPase in ER and is usually applied as an ER stress activator^46^.

Transient inhibition of BiP was carried out by transferring cells with 50 nM BiP siRNA (CGA GUG ACA GCU GAA GAC AAG GGU A) or the scrambled siRNA as control (Genepharma, Shanghai, China) using Lipofectamine 2000 (Invitrogen) according to the manufacturer’s instructions. Then the cells were harvested and re-seeded for further experimental utilizations.

### MN scoring

MN were measured using the cytokinesis block technique described by our previous work^47^. MN were scored in at least 500 binucleated cells and the MN yield, Y_MN_, was calculated as the ratio of the number of MN to the scored number of binucleated cells.

### Apoptosis assay

Apoptosis was assessed by flow cytometry (Guava EasyCyte System, Guava Technologies, Hayward, CA, USA) using the Guava Nexin Reagent (Guava Technologies). Briefly, ICCM treated bystander cells were collected, washed with 1 × PBS, treated with Guava ViaCount Cell Dispersal Reagent at 37 °C for 5 minutes, then resuspended in culture medium and centrifuged at 300 g for 6 min. The cell pellet was suspended with FBS at concentration of 1 × 10^6^/mL and stained with the Guava Nexin reagent (Guava Technologies) according to the manufacturer’s instructions. Ten thousand cells from each sample were analyzed by the cytometry.

### Western blot assay

Antibodies including BiP, IRE1α, PERK, CHOP and p-eIF2α (Ser-51) were purchased from Cell Signaling Technology (Danvers, MA, USA). Tubulin antibody and the HRP-conjugated secondary antibodies were purchased from Beyotime Biotechnology (Nantong, Jiangsu, China). Cell lysates were prepared as described before^45^. An equal amount of total protein was subjected to 10% SDS-PAGE and transferred to a PVDF membrane (Millipore, Bedford, MA, USA). Membranes were probed with primary antibodies as indicated. Tubulin was used for the loading control.

### RNA extraction and reverse transcriptional polymerase chain reaction (RT-PCR)

Total RNA was extracted from cells using Trizol reagent (OMEGA, Norcross, GA, USA) according to the manufacturer’s instruction, then 1.0 μg RNA for each sample was reverse-transcribed using a PrimeScript™ RT reagent Kit (Takara Biotechnology, Dalian, Liaoning, China) and 10% of the reverse transcriptional reaction was used in PCR amplification to detect the fragments of XBP-1 and β-actin, respectively. The PCR products were visualized after electrophoresis in 1.8% agarose gels by ethidium bromide staining. Relative amounts of amplified DNA were semi-quantified using Multi Gauga Ver 2.2 image analyzing software. The sequences of the primers of XBP-1 and β-actin are listed in Table 2.

### Statistical analysis

The data were expressed as means ± S.E. of at least three independent experiments. The significance of differences in data of different groups were appropriately determined by the unpaired Student’s *t*-test at *P* < 0.05.

## Additional Information

**How to cite this article:** Xie, Y. *et al*. Protective effect of mild endoplasmic reticulum stress on radiation-induced bystander effects in hepatocyte cells. *Sci. Rep.* **6**, 38832; doi: 10.1038/srep38832 (2016).

**Publisher's note:** Springer Nature remains neutral with regard to jurisdictional claims in published maps and institutional affiliations.

## Figures and Tables

**Table 1:** Fraction of XBP-1 mRNA splicing as a percentage of total XBP-1 mRNA in bystander HL-7702 cells treated with Thapsigargin, *BiP* siRNA, or their combination before receiving ICCM from normoxic and hypoxic HepG2 cells. Data were calculated from the results in Fig. 5a and b.

Treatment	O_2_- Irradiated Conditioned Medium		N_2_- Irradiated Conditioned Medium	
	0 Gy	3 Gy	0 Gy	3 Gy
Control	0	0	0	0
Thapsigargin	0.266 ± 0.0013^a^	0.270 ± 0.0052^a^	0.396 ± 0.0026^a^	0.396 ± 0.0014^a^
Control siRNA	0	0	0	0
BiP siRNA	0	0	0	0
BiP siRNA+ Thapsigargin	0.685 ± 0.0074^b^,^c^	0.658 ± 0.0030^b^,^c^	0.688 ± 0.0052^b^,^c^	0.674 ± 0.0072^b^,^c^

^a^*P* < 0.001 compared to control receiving the same ICCM.

^b^*P* < 0.001 compared to *BiP* siRNA receiving the same ICCM.

^c^*P* < 0.001 compared to Thapsigargin receiving the same ICCM.

**Table 2:** Primer sequences for XBP-1 and β-actin.

Primer	Sequence (5′ to 3′)	Length
XBP-1 Forward primer	5′-CCTTGTAGTTGAGAACCAGG-3′	442 bp
Reverse primer	5′-GGGGCTTGGTATATATGTGG-3′	416 bp
β-actin Forward primer	5′-CCTGGCACCCAGCACAAT-3	144 bp
Reverse primer	5′-GGGCCGGACTCGTCATAC-3′	144 bp
